# Understanding our thoracic surgery workforce: Who, what, and where we practice

**DOI:** 10.1016/j.xjon.2025.08.016

**Published:** 2025-09-18

**Authors:** Jacob Daniel, Malcolm DeCamp, Jennifer Romano, Betty Tong, Michael Moulton, John Mitchell, Carl Backer, Joseph Cleveland, David T. Cooke, Raphael Kella, Joseph Coselli, Jessica Donington, Stephanie Fuller, Adil Husain, Chris Malaisrie, Sandra Starnes, John Stulak, Cameron Wright, Michael Mack, Tom C. Nguyen

**Affiliations:** a Division of Cardiothoracic Surgery, Department of Minimally Invasive Cardiac Surgery, Miami Cardiac and Vascular Institute, Miami, Fla; b Division of Cardiothoracic Surgery, UW Hospitals and Clinics, Madison, Wis; c Department of Congenital Heart Surgery, University Of Michigan, Ann Arbor, Mich; d Department of Cardiovascular and Thoracic Surgery, Duke University, Durham, NC; e Division of Cardiothoracic Surgery, University of Nebraska Medical Center, Omaha, Neb; f Department of Cardiothoracic Surgery, University of Colorado Hospital, Aurora, Colo; g Department of Surgery, Department of Pediatrics, Cincinnati Children's Hospital Medical Center, Winnetka, Ill; h Division of General Thoracic Surgery, University of California, Sacramento, Calif; i Department of Internal Medicine, Florida Atlantic University, Boca Raton, Fla; j Department of Surgery, Baylor College of Medicine, Houston, Tex; k Section of Thoracic Surgery, University of Chicago Medicine, Chicago, Ill; l Division of Cardiothoracic Surgery, The University of Pennsylvania, Philadelphia, Pa; m Section of Pediatric Cardiothoracic Surgery, University of Utah Hospital, Salt Lake City, Utah; n Division of Cardiothoracic Surgery, Northwestern Memorial Hospital, Chicago, Ill; o Division of Cardiothoracic Surgery, University of Cincinnati Medical Center, Cincinnati, Ohio; p Division of Cardiothoracic Surgery, Mayo Clinic, Rochester, Minn; q Department of Thoracic Surgery, Harvard University, Boston, Mass; r Department of Cardiothoracic Surgery, Baylor Scott & White Health, Plano, Tex

**Keywords:** cardiothoracic surgery, gender diversity, healthcare policy, Maintenance of Certification, subspecialization, workforce trends

## Abstract

**Objective:**

The makeup of the thoracic surgical workforce can influence policy, training, and certification, but it is not well defined. Using data from the American Board of Thoracic Surgery, this study explored practice-based demographics concerning geography, gender, age, subspecialty, and university affiliation.

**Methods:**

American Board of Thoracic Surgery Diplomates taking the 10-year Maintenance of Certification examination opted for the cardiac, general thoracic, cardiothoracic, or congenital modular exam. Using module selection as a surrogate for the examinee's predominant clinical practice, we explored the relationship regarding type of practice, geography (metropolitan vs other), gender, age, and university affiliation.

**Results:**

A total of 2273 American Board of Thoracic Surgery Diplomates took the Maintenance of Certification exam from 2018 to 2024. Adult cardiac surgery was the predominant subspecialty (46%), followed by cardiothoracic (24%), general thoracic (22%), and congenital surgery (8%). Significant gender disparity persisted, with women constituting 7% of certified Diplomates and 5% of adult cardiac surgeons. Mean ages ranged from 58.0 years (general thoracic) to 63.3 years (cardiothoracic), with younger surgeons trending toward specialized practices (cardiac *P =* .01, congenital *P =* .04). Most surgeons practiced in metropolitan areas (80%), particularly congenital surgeons (96%). Surgeons practicing in university (47%) and nonuniversity settings (53%) were nearly evenly distributed.

**Conclusions:**

Thoracic surgery is increasingly subspecializing, with younger surgeons choosing cardiac, general thoracic, or congenital surgery modular Maintenance of Certification exams. The percentage of female Diplomates remains low. Maintenance of Certification exam-eligible diplomates constitute a predominantly older workforce with noticeable urbanization. Understanding our workforce provides important insight for American Board of Thoracic Surgery certification, the development of training paradigms, and anticipating workforce needs.

**Figure:**
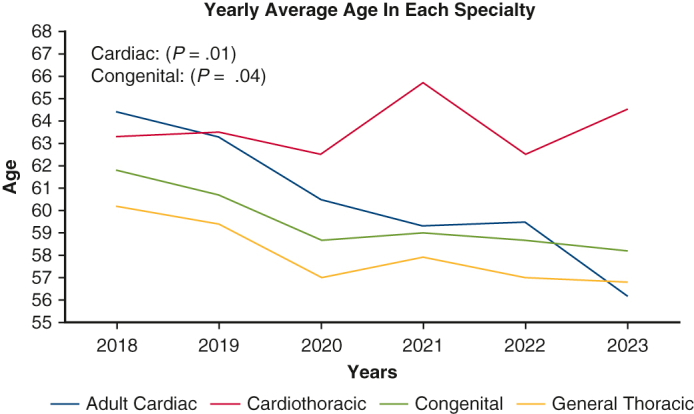
Yearly average age in each specialty: The average age of surgeons in subspecialties decreased over time.

**Central Message**

Younger thoracic surgeons increasingly subspecialize. Gender disparities prevail, and the workforce is both aging and urbanizing, demonstrating the volatility of the thoracic workforce composition.

**Perspective**

The increasing subspecialization of young surgeons and the prevalence of cardiothoracic [hybrid] practice among older surgeons have significant implications for future workforce sustainability and training. Moreover, the mean age of the thoracic workforce is increasing, placing the specialty at risk for gaps in surgical expertise as the current workforce approaches retirement age.

Cardiothoracic surgery is experiencing a period of rapid transformation, driven by increasing procedural complexity and integration of innovative technologies, including, but not limited to, transcatheter interventions, complex aortic procedures, robotic approaches, personalized treatments of cancer, and advanced thoracic techniques. As the specialty evolves, it is critical to understand the composition, distribution, and characteristics of the current workforce to inform policies related to training, certification, and future workforce planning. Gender distribution within the field remains an important area of focus, with persistent disparities that warrant continued evaluation. By using data from the American Board of Thoracic Surgery (ABTS), this study explores key demographic and practice-related trends across geography, gender, age, and potential university affiliation. This analysis aims to provide a comprehensive overview of the contemporary cardiothoracic surgical workforce and to identify patterns that may inform strategic planning and support future considerations around workforce development and certification frameworks.

## Material and Methods

In January 2008, the ABTS transitioned from a traditional recertification model to a Maintenance of Certification (MOC) process, designed to ensure that board-certified thoracic surgeons continue to meet high standards of clinical care throughout their careers. The MOC process operates on a 10-year cycle with Diplomates required to pass a secure online examination and submit documentation related to current practice. When registering for the MOC examination, Diplomates have the option of choosing a cardiac, general thoracic, combined cardiothoracic (hybrid practice), or congenital modular examination. Concerning nonmetropolitan/nonuniversity practice settings, these are mostly composed of community-based hospitals that provide basic cardiothoracic surgical services; some are academically affiliated and provide a wide spectrum of cardiothoracic surgical services with regard to complexity and subspecialization. Module selection was used as a surrogate for the examinee's predominant clinical practice.

Diplomates taking the 10-year MOC from 2018 to 2024 were included in the study. There were no exclusions due to missing or incomplete data from the National Provider Identifier registry, web searches, or ABTS. Age, gender, city and state of primary practice, metropolitan status (US Census Bureau definition of regions with a population of 50,000 residents or more),^1^ and university affiliation were obtained for each Diplomate. To explore the relationship of demographic data with respect to type of practice, data regarding age, gender, city, and state of practice were obtained from the publicly available National Plan and Provider Enumeration System National Provider Identifier Registry,^2^ whereas geographic categorization (metropolitan vs other) and university affiliation were determined by referencing the US Census Bureau and web searches, respectively.

Statistical analyses consisted of descriptive statistics to summarize the data, proportions to represent categorical distributions, and linear regression to evaluate trends over time. This research used publicly available, deidentified data; therefore, it was exempt from Institutional Review Board approval.

## Results

Between 2018 and 2024, a total of 2273 Diplomates took the 10-year MOC examination with the following modules: adult cardiac, general thoracic, cardiothoracic, and congenital surgery.

### Diplomate Distribution

Among Diplomates selecting adult cardiac or general thoracic MOC pathways, the mean ages were 61.5 and 58.4 years, with 95% and 87% male representation, respectively. These groups also demonstrated high urban concentration (80% and 78%) and an approximate 50:50 division between university and nonuniversity institutions. The cardiothoracic MOC group mirrored these trends, except for a higher proportion practicing in nonuniversity hospitals (70%). In contrast, 73% of congenital MOC examinees reported affiliation with university hospitals (Table 1).

**Table 1:** Diplomate distribution in 2018 to 2024

Specialty	Average age, y	Male	Female	Metropolitan	Nonmetropolitan	University hospital	Nonuniversity hospital
Adult Cardiac	61.5	95% (987)	5% (55)	80% (830)	20% (212)	49% (509)	51% (533)
Cardiothoracic	62.4	96% (532)	4% (25)	79% (440)	21% (117)	30% (166)	70% (391)
Congenital	60	92% (164)	8% (15)	96% (171)	4% (8)	73% (131)	27% (48)
General Thoracic	58.4	87% (431)	13% (64)	78% (388)	22% (107)	55% (273)	45% (222)

**Figure 1:**
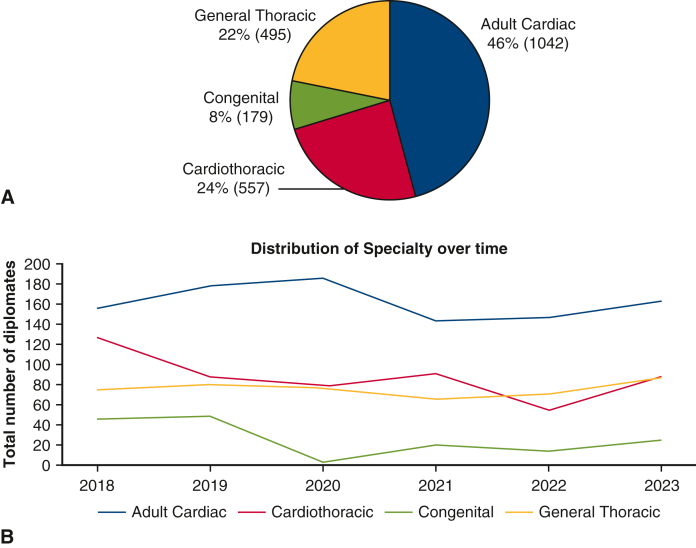
Yearly distribution of diplomates in each specialty.

The workforce consists primarily of cardiac surgeons. Approximately half of all certified surgeons selected the cardiac MOC pathway (46%). The thoracic and cardiothoracic MOC tracks were chosen by 22% and 24% of candidates, respectively. Congenital surgery was selected by only 8% of Diplomates (Figure 1, *A*).

A chi-square test for independence was performed to examine the relationship between MOC specialty selection and year. The association between year and specialty was statistically significant (chi-square = 76.98, *df* = 15, *P < .*001), indicating notable shifts in specialty distribution from 2018 to 2023 (Figure 1, *B*). Adult cardiac surgery consistently remained the predominant specialty, peaking in 2020 (n = 186) but subsequently declining in 2021 (n = 143), followed by a modest recovery in 2023 (n = 163). Cardiothoracic surgery demonstrated a downward trend, declining from 127 Diplomates in 2018 to 55 Diplomates in 2022, but showed some rebound in 2023 (n = 88). Congenital surgery showed an initial significant decline from 46 Diplomates in 2018 to only 3 Diplomates in 2020, but also demonstrated recovery, increasing to 25 Diplomates by 2023. In contrast, general thoracic surgery showed a consistent incremental increase from 75 Diplomates in 2018 to 87 Diplomates in 2023, reflecting steady growth in surgeon preference toward focused general thoracic practice.

### Gender Representation

**Figure 2:**
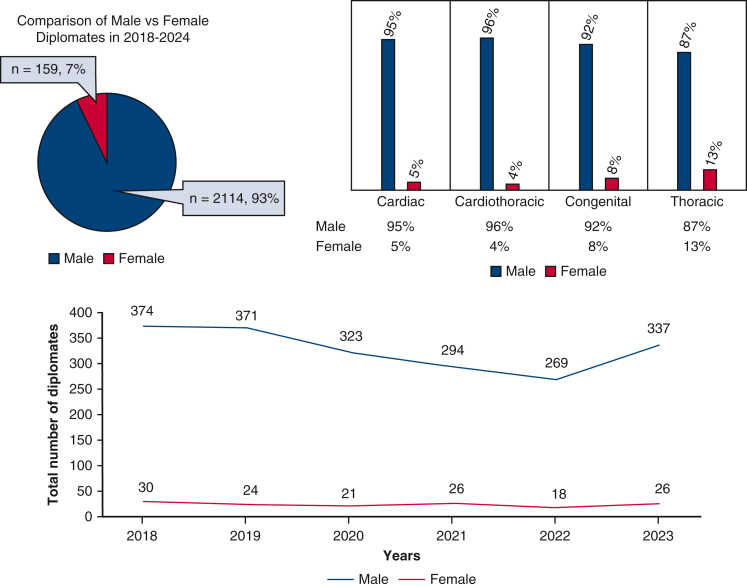
Gender representation of diplomates 2018 to 2024.

A pronounced gender imbalance was noted across all subspecialties. Of the 2273 total Diplomates, 93% (n = 2114) were male and only 7% (n = 159) were female (Figure 2). The greatest disparity was in cardiothoracic surgery, where women represented 4%. Cardiac surgery had a 5% female representation, and congenital surgery had 8%. General thoracic surgery had the highest female participation at 13%.

### Age Distribution

The mean age of the entire cohort was 60.2 years. Mean age at MOC examination varied by specialty (Table 2). Cardiothoracic surgeons were generally the oldest (mean age, 63.3 years), followed by adult cardiac (mean age, 60.3 years), congenital (mean age, 59.4 years), and general thoracic surgeons (mean age, 58.0 years). These averages remained stable year over year with minor variations.

**Table 2:** Average age per specialty per year

Specialties	2018	2019	2020	2021	2022	2023	2024	Mean age, y	*P* value
Cardiac	64.4	63.3	60.5	59.3	59.5	56.2	59.4	**60.3**	**.01**
Cardiothoracic	63.6	63.5	62.5	65.7	62.5	64.5	61.3	**63.3**	**.57**
Congenital	61.8	60.7	58.7	59	58.7	58.2	59.2	**59.4**	**.04**
Thoracic	60.2	59.4	57	57.9	57	56.8	57.8	**58.0**	**.06**

Their significance is to emphasize the mean age and *P* values as the central focus of the table.

**Figure 3:**
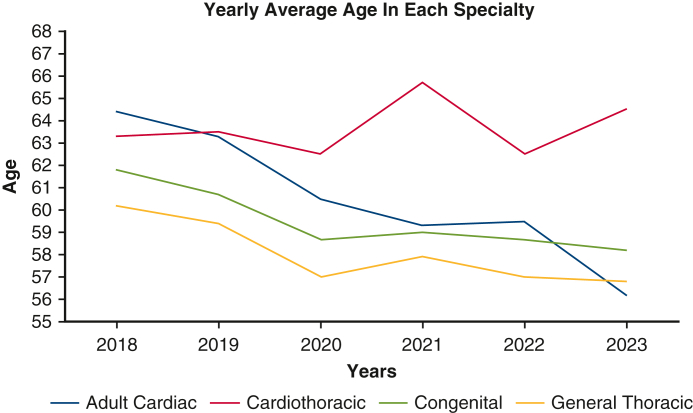
Average age per specialty per year.

Linear regression showed that cardiac (*P =* .01) and congenital (*P =* .04) subspecialties experienced statistically a significantly younger average age of surgeons over time, suggesting that younger surgeons are gradually gravitating toward more focused, rather than combined, specialty tracks (Figure 3).

### Practice Geography by Subspecialty

**Figure 4:**
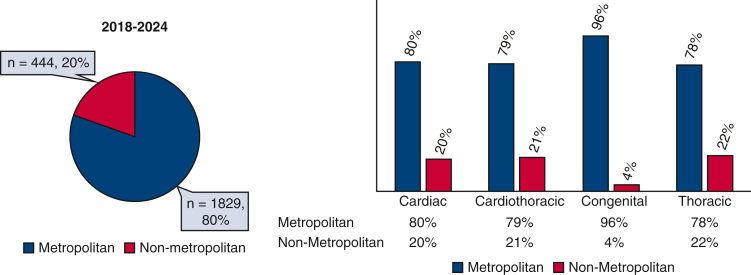
Distribution of specialty by metropolitan and nonmetropolitan cities.

A majority of certified surgeons (n = 1829; 80%) practiced in metropolitan areas. This trend was most pronounced in congenital surgery, where 96% were based in urban settings, with only 8 surgeons working in rural areas (Figure 4).

### Institutional Affiliation by Subspecialty

**Figure 5:**
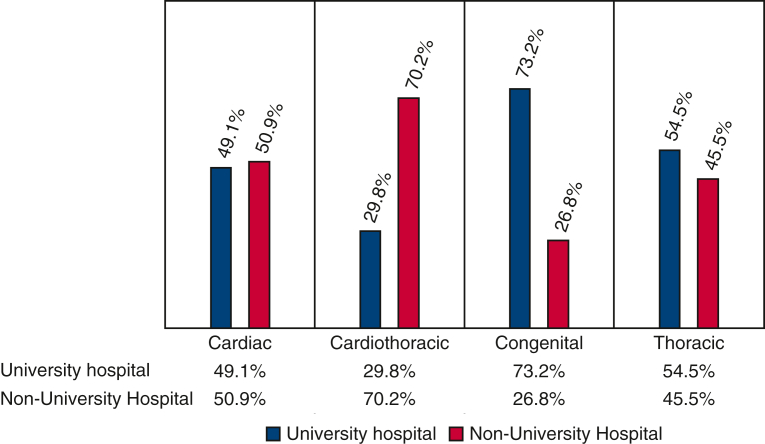
Proportion of each specialty practicing in university and nonuniversity.

Specialty-specific distributions varied. A total of 70.2% of cardiothoracic Diplomates practiced in nonuniversity settings, and 29.8% practiced in university hospitals. Among congenital surgeons, 73.2% practiced in university hospitals and 26.8% practiced in nonuniversity settings. Cardiac surgeons were distributed as 49.1% in university hospitals and 50.9% in nonuniversity hospitals, whereas thoracic surgeons were distributed as 54.5% in university hospitals and 45.5% in nonuniversity hospitals (Figure 5).

## Discussion

This research offers valuable perspectives on the current cardiothoracic surgery workforce in the United States, emphasizing significant demographic changes, geographic distribution, and trends in subspecialization. Important highlights of the study include the following: Our specialty consists of a predominantly older workforce with noticeable urbanization. The mean age of the cohort was 60.2 years. With respect to specialty, our workforce consists primarily of cardiac surgeons (46%). There remains a pronounced gender imbalance, with the workforce being 93% male. General thoracic surgery had the highest female representation at 13%. Last, younger surgeons tend to subspecialize in adult cardiac, general thoracic, or congenital cardiac surgery, whereas older surgeons tend to practice cardiothoracic surgery.

This study cohort is representative of the cardiothoracic surgical workforce and aligns with prior national estimates. Between 2018 and 2024, a total of 2273 ABTS Diplomates completed the 10-year MOC examination, comprising approximately 61% of the 3680 valid ABTS certificate holders and reflecting a substantial portion of actively practicing board-certified cardiothoracic surgeons in the United States. These findings are consistent with national workforce estimates of 4000 to 4200 practicing surgeons.^3^^,^^4^ Moreover, our results parallel with the STS 2024 Workforce and Compensation Survey, which reported 48% of respondents in cardiac surgery, 23% in general thoracic, and 18% in hybrid practice.^5^ This concordance supports the validity of the MOC cohort as a proxy for workforce assessment.

The mean age of practicing board-certified thoracic surgeons currently stands at 60.2 years, reflecting an increasingly aged workforce. This finding is consistent with prior workforce surveys, where the median or mean age has steadily trended upward. In the Society of Thoracic Surgeons (STS) 2019 Workforce Report, the median age of thoracic surgeons was reported as 56 years, highlighting early concerns about the specialty's aging demographic and future workforce sustainability.^6^ Likewise, the STS/American Association for Thoracic Surgeons Snapshot 2010 Survey by Shemin and Ikonomidis^7^ found the median age to be 52.9 years, already signaling a gradual demographic shift toward older practitioners within the specialty. When compared with other specialties, the thoracic surgery workforce appears significantly older. According to recent studies, the mean age of practicing general surgeons is reported to be approximately 49 to 51 years,^8^ whereas neurosurgeons have a mean age of 50.6 years and orthopedic surgeons have an age of approximately 49.5 years.^8^^,^^9^ In contrast, internal medicine physicians have an average age of approximately 48 years.^10^ Collectively, these comparisons underscore that thoracic surgery is uniquely burdened by a disproportionately older practitioner base relative to both surgical and nonsurgical fields, largely due to the length of training required to be board-eligible. The implications are profound: A large proportion of the workforce is approaching retirement age, risking potential gaps in surgical expertise, case volume management, and innovation adoption.

An important finding in our study reveals increasing specialization in our field. Younger surgeons were more likely to select cardiac, general thoracic, or congenital MOC examinations. As the current workforce ages, the proportion of surgeons practicing both cardiac and thoracic surgery is likely to decline. This aligns with previous data showing younger average ages in congenital and cardiac subspecialists compared with those maintaining a general cardiothoracic designation.^11^ The shift also reflects growing clinical and technical complexity within each subspecialty. Innovations such as transcatheter valve interventions (eg, transcatheter aortic valve implantation, transcatheter edge-to-edge repair), robotic-assisted thoracic surgeries, and interventional approaches to congenital cardiac surgery demand deeper, more focused expertise. These demands could make more general training less practical for recent graduates, highlighting the necessity for specialized training programs.^11^^,^^12^ Prior workforce reports have observed similar shifts. Shemin and Ikonomidis^7^ noted that advancing surgical complexity, length of training, and early subspecialization were reshaping the identity of the thoracic surgery workforce as early as 2010. Likewise, a 2022 congenital surgery workforce survey emphasized the need for targeted workforce planning due to the increasing alignment of training with focused subspecialty practice.^13^ As this trend continues, understanding patterns of MOC pathway selection will be critical for aligning fellowship capacity, certification policy, and regional access needs across the subspecialties. This distinct trend toward subspecialization, where younger Diplomates disproportionately select cardiac, thoracic, or congenital MOC modules, has significant implications for future workforce sustainability and training paradigms.

Gender disparities remain significant within the thoracic surgery workforce, with women representing only 7% of the total workforce, closely mirroring the 8% female representation reported in the recent STS Workforce surveys.^5^ Female surgeons predominantly practice in general thoracic surgery (13%), whereas cardiothoracic surgery has the lowest female representation (4%). The proportion of women practicing thoracic surgery was approximately 3 times higher than those practicing cardiothoracic surgery. This finding highlights a disconnect between current workforce composition and trends observed at the medical school level. Unfortunately, because the number of female diplomates was low (7% of the sample size), it was difficult to extrapolate any significant trends regarding female surgeons practicing at university versus nonuniversity hospitals. Although a greater number of women are enrolling in medical schools compared to men, they continue to be significantly underrepresented in the specialty of cardiothoracic surgery. This inconsistency may underscore persistent systemic obstacles, such as gaps in mentorship, restricted leadership prospects, duration of training, and institutional biases, as detailed in recent reports from Women in Thoracic Surgery.^14^^,^^15^ Philosophically and ethically, the field must address these disparities to ensure equitable representation. Encouragingly, the slight upward trend among younger female surgeons point to gradual progress through targeted recruitment and retention strategies.

Comparing our findings with earlier workforce studies, particularly Shemin and Ikonomidis,^7^ reveals the urbanization of our workforce. Previously, urban settings comprised 56% of thoracic surgical practice, significantly lower than our finding of 80%. Specifically, congenital surgeons have become even more concentrated in urban areas, with their percentage rising from 79%^7^ to 96% in our current research. Conversely, the prevalence of urban practice among adult cardiac and general thoracic surgeons has significantly risen from the previously documented rates of 51% to 62%, respectively, indicating a clear shift toward urbanization. Eberth and colleagues^3^ noted significant geographic barriers, noting that 75% of counties lacking thoracic surgeons were rural, with disparities widening over recent years. Collectively, these comparisons confirm a steady urbanization and subspecialization of thoracic surgical practice.^6^^,^^7^ Recent reviews on thoracic surgical practice in the United States, such as by Byrd and colleagues,^4^ noted approximately 530,000 thoracic surgical procedures annually performed predominantly by approximately 4000 cardiothoracic surgeons, with most operating in hospital-based (44.8%) or academic settings (33.6%). This aligns closely with our findings of nearly equal distribution between university (47%) and nonuniversity (53%) institutions.^4^

### Limitations

This study is subject to several limitations. The data are limited to Diplomates taking the 10-year ABTS MOC examination between 2018 and 2024. As a result, this study inherently underrepresents early-career surgeons not yet eligible for recertification. Regarding gender demographics, we found 7% of Diplomates taking the MOC exams were female. It is likely that this percentage will increase in the near future, given that more than 20% of cardiothoracic surgery trainees are female,^16^ and in 2024, more than 30% of cardiothoracic surgery training applicants were female. The MOC modular exam serves as a proxy for clinical practice patterns but may not fully reflect hybrid or evolving practices, particularly in nonacademic settings. We could not find direct validation for this assumption from prior publications. This could be grounds for further investigation, either by survey or concordance data. Additionally, the study is limited to MOC exam-takers, excluding early-career surgeons and those recertifying via alternate pathways. This creates a selection bias toward older, academically inclined, and board-compliant individuals. Finally, surgeons who change their primary practice focus between certification cycles, such as from cardiac to cardiothoracic or thoracic surgery, are not captured in their new specialty until the next recertification, leading to delayed recognition of subspecialty transitions.

## Conclusions

This study highlights 3 critical dynamics shaping the contemporary thoracic surgical workforce. First, subspecialization is accelerating, particularly among younger surgeons, with fewer Diplomates pursuing combined cardiothoracic certification and more selecting focused MOC modules in cardiac, thoracic, or congenital cardiac surgery. Second, gender disparities remain pronounced, with women comprising a small but gradually increasing proportion of the workforce, especially in academic settings. Third, the specialty consists of a predominantly older workforce with noticeable urbanization, raising concerns about workforce sustainability and timely access to specialized care for nonurban communities. These trends highlight the necessity to reevaluate training frameworks, encourage inclusiveness, and facilitate focused workforce renewal.

### Webcast

You can watch a Webcast of this AATS meeting presentation by going to: https://www.aats.org/resources/understanding-our-thoracic-sur-10049.

**Figure:**
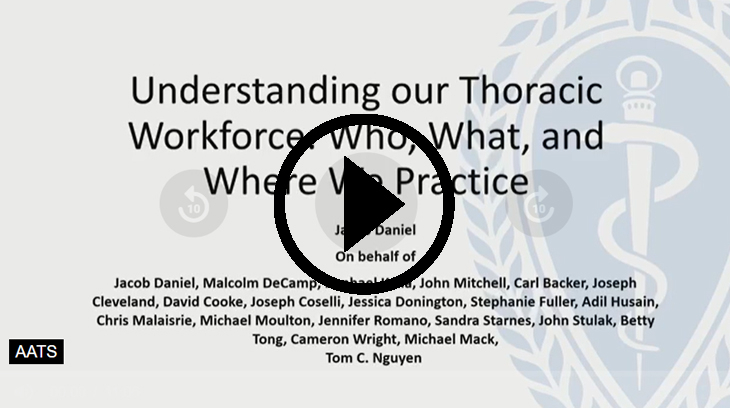

### Audio

You can listen to the discussion audio of this article by going to the supplementary material section below.

## Conflict of Interest Statement

The authors reported no conflicts of interest.

The *Journal* policy requires editors and reviewers to disclose conflicts of interest and to decline handling or reviewing manuscripts for which they may have a conflict of interest. The editors and reviewers of this article have no conflicts of interest.

## Supplementary Data

### Audio

Audio 1 Please note there is an audio/video file to be added as supplementary material with the below caption: This is an audio recording of the presentation of the associated abstract and discussion. The abstract presentation ends at 6:06 and the discussion begins at 6:10.

Video 1 Xxx. Audio presentation of the associated Abstract and Discussion. Video available at: https://www.jtcvs.org/article/S2666-2736(25)00304-3/fulltext.
